# Inhibition of DKC1 induces telomere-related senescence and apoptosis in lung adenocarcinoma

**DOI:** 10.1186/s12967-021-02827-0

**Published:** 2021-04-20

**Authors:** Guangyan Kan, Ziyang Wang, Chunjie Sheng, Chen Yao, Yizhi Mao, Shuai Chen

**Affiliations:** State Key Laboratory of Oncology in South China, Collaborative Innovation Center for Cancer Medicine, Sun Yat-sen University Cancer Center, Guangzhou, 510060 Guangdong People’s Republic of China

**Keywords:** Lung adenocarcinoma, DKC1, Telomere, Cell senescence

## Abstract

**Background:**

Lung cancer is one of the most widely spread cancers in the world and half of the non-small cell lung cancers are lung adenocarcinoma (LUAD). Although there were several drugs been approved for LUAD therapy, a large portion of LUAD still cannot be effectively treated due to lack of available therapeutic targets. Here, we investigated the oncogenic roles of DKC1 in LUAD and its potential mechanism and explored the possibility of targeting DKC1 for LUAD therapy.

**Methods:**

The Gene Expression Omnibus (GEO) and The Cancer Genome Atlas Program (TCGA) databases were used to examine the *DKC1* transcript levels. Gene expression with clinical information from tissue microarray of LUAD were analyzed for associations between DKC1 expression and LUAD prognosis. In addition, loss- and gain-of-function assays were used for oncogenic function of DKC1 both in vitro and in vivo.

**Results:**

DKC1 is overexpressed in LUAD compared with adjacent normal tissues. High expression of DKC1 predicts the poor overall survival. DKC1 knockdown in LUAD cell lines induced G1 phase arrest and inhibited cell proliferation. Ectopic expression of DKC1 could rescue the growth of LUAD cell lines. In addition, the abundance of *DKC1* is positively correlated with *telomerase RNA component (TERC)* and *telomerase reverse transcriptase (TERT)* levels in LUAD. DKC1 downregulation resulted in decreased *TERC* expression, reduced telomerase activity and shorten telomere, and thus eventually led to cell senescence and apoptosis.

**Conclusions:**

Our results show that high DKC1 expression indicates poor prognosis of LUAD and DKC1 downregulation could induce telomere-related cell senescence and apoptosis. This study suggests that DKC1 could serve as a candidate diagnostic biomarker and therapeutic target for LUAD.

**Supplementary Information:**

The online version contains supplementary material available at 10.1186/s12967-021-02827-0.

## Background

Lung cancer is the leading cause of cancer death worldwide [1]. Lung adenocarcinoma (LUAD) is currently the most common type of lung cancer, which counts for about half of all non-small cell lung cancers [2]. In the past decades, there are several diver genes for LUAD having been identified and targeted therapies against them have achieved remarkable success. Inhibitors against epidermal growth factor receptor (EGFR), anaplastic lymphoma kinase (ALK), BRAF V600E mutation and mesenchymal-to-epithelial transition (MET) Exon 14 skipping have been approved by FDA for precision treatment of LUAD [3, 4]. Despite these progressions, there is still large portion of LUAD without available therapeutic target.

The dyskerin pseudouridine synthase 1 (DKC1) gene which encodes dyskerin was first identified in dyskeratosis congenita (DC) [5, 6]. Dyskerin is one component of telomerase ribonucleoprotein that associates with three highly conserved proteins (NOP10, NHP2 and GAR1) and binds directly to telomerase RNA component (TERC) to maintain TERC stability, telomerase stability and telomere length [7, 8]. Recent reports showed that dysregulated expression of DKC1 in various human cancers alters cancer cell growth or metastasis and is associated with patient prognosis [9–11]. In addition, the pyrazofurin (PF) was identified as a potent DKC1 inhibitor [12]. However, whether DKC1 promotes cancer cell growth in lung cancer and whether DKC1′s oncogenic function is dependent on the regulation of telomere remain largely unknown.

This study aimed to investigate DKC1 expression in LUAD and to examine the relation between DKC1 expression and patient prognosis. Moreover, this study has explored the oncogenic function of DKC1 both in vitro and in vivo and the potential mechanism of DKC1 promoting tumor progression.

## Methods

### Cell culturing and human tissue microarray

A549, PC-9 and NCI-H1299 cells were obtained from American Type Culture Collection (ATCC) and maintained by following the culture instructions of ATCC. Human lung adenocarcinoma tissue microarray (HLugA020PG01) was obtained from Shanghai Outdo Biotech Company. There were 84 lung adenocarcinoma tissues and 70 adjacent normal tissues. The clinical data of patients were showed in Additional file 1: Table S1. And the association of DKC1 expression and patient’s survival was analyzed.

### DKC1 expression in LUAD samples from GEO and TCGA databases

The mRNA level of *DKC1* in 83 LUAD tissues and paired adjacent normal tissues from the Gene Expression Omnibus (GEO) database (GSE75037) was examined. GEO databases (GSE12930 and GSE102016) were used to examine the mRNA level of *Dkc1* in inflammatory non-transformed lung. (http://www.ncbi.nlm.nih.gov/geo/). GSE12930 shows gene expression in smoking induced inflammation non-transformed mouse lung [13]. GSE102016 shows gene expression in non-transformed lung from phytol ameliorated benzo(a)pyrene (B[a]P) induced lung cancer mouse model with lipopolysaccharides (LPS) induced inflammation [14]. In addition, we searched The Cancer Genome Atlas Program (TCGA) database (https://cancergenome.nih.gov/) to examine *DKC1* expression in LUAD tissues. There were 483 tumor tissues and 59 adjacent normal tissues. The extracted data were normalized and processed by log2 transformation.

### Generation of stable knockdown or overexpression cells

shRNA for DKC1 (shDKC1-1, -2) was cloned into lentiviral pLKO.1 construct (Sigma-Aldrich, St. Louis, USA). The sequence of the shDKC1-1 is CCGGGCTCAGTGAAATGCTGTAGAACTC GAGTTCTACAGCATTTCACTGAGCTTTTTG and the sequence of the shDKC1-2 is CCGGTAT GTTGACTACAGTGAGTCTCTCGAGAGACTCACTGTAGTCAACATATTTTTG. The coding sequence of DKC1 was cloned into lentiviral pCDH-EF1-T2A-zeocin vector (System Bioscience, CA, USA). The virus particles were produced according to the lentivirus packaging protocol of Addgene. After shRNA lentivirus infection of lung adenocarcinoma cells lines (A549 and PC-9), puromycin (Thermo Fisher Scientific, Waltham, USA) selection was applied for at least 7 days to obtain stable knockdown cells. The overexpression lentivirus infected DKC1 stable knockdown cells or NCI-H1299 cells were selected with zeocin (Thermo Fisher Scientific, Waltham, USA) for at least 7 days to obtain ectopically expressed cells.

### Cell proliferation, cell cycle and cell apoptosis assay

For cell growth curve, 1500 cells were plated in one well of 96-well plate with at least 3 repeats for each cell and Cell Counting Kit-8 (CCK-8, Dojindo, Tokyo, Japan) was used to do continuous detection for 4–5 days. For colony formation assay, 1000 cells were plated in one well of 6-well plate with at least 3 repeats for each cell and 7–9 days later were stained with crystal violet. Image J was used for colony number counting. For pyrazofurin (SML1502, Sigma-Aldrich, St. Louis, USA) affecting cell growth, 1500 cells were plated in one well of 96-well plate, with at least 3 repeats for each condition and the next day pyrazofurin or vehicle was added. CCK-8 was used to do the continuous detection for 4–5 days. For cell cycle, one million of cells were harvested and fixed using pre-cold 70% ethanol at 4 ℃, overnight. The next day, centrifuge and discard the supernatant, and wash the cells with PBS twice. Then add the propidium iodide (PI) staining solution (C1052, Beyotime Biotechnology, shanghai, China) and incubate cells at room temperature for 15 min. Then go for the flow cytometric analysis. For cell apoptosis, the DKC1 knockdown A549 and PC-9 cells and control cells were harvested for apoptosis markers (P21 and γH2A.X) analysis using western blot.

### Western blot

The cells were harvested and lysed with RIPA lysis buffer (9806, Cell Signaling Technology, Boston, USA) containing protease inhibitors cocktail (B14002, Bimake, Houston, USA) and phosphatase inhibitor (B15001, Bimake, Houston, USA). After centrifugation, the protein concentration was measured using BCA protein assay kit (23,225, Thermo Fisher, Waltham, USA) and 30 μg lysates protein were subjected to 12% SDS-PAGE. The following antibodies were used for western blot: DKC1 (sc-373956, Santa Cruz Biotechnology, CA, USA), P21 (sc-6246, Santa Cruz Biotechnology, CA, USA), γH2A.X (2595, Cell Signaling Technology, Boston, USA), GAPDH (RM2002, Beijing Ray Antibody Biotech, China).

### Quantitative PCR-based telomerase repeated amplification protocol

The Telomerase Repeated Amplification Protocol (TRAP) is one sensitive telomerase activity detection assay. The quantitative PCR-based TRAP assay (qTRAP) is a more convenient measurement methods [15]. DKC1 stable knockdown A549 and PC-9 cells or control cells were harvested and lysed on ice for 30 min using NETN buffer (40 mM Tris–HCl, pH 8.0, 100 mM NaCl, 0.5% NP40, 1 mM EDTA, 10% glycerol, 1 mM DTT, and protease inhibitors in nuclease-free water). 5000, 500 or 50 cell lysates were incubated in quantitative RT-PCR buffer (1708884AP, Bio-Rad, Hercules, USA) with TS primers and ACX primers at 30℃ for 30 min. After the primer extension step, then do PCR following the steps: 90 s at 94 °C; 40 cycles of 30 s at 94 °C and 60 °C each; 45 s at 72 °C. The products of qTRAP (5000 cells) were visualized on a 10% non-denaturing acrylamide gel.

- TS primer: 5′-AATCCGTCGAGCAGAGTT-3′
- ACX primer: 5′-GCGCGG(CTTACC)3CTAACC-3′

### Telomere length measurements

The mean telomere length was measured by a quantitative PCR-based technique [16] in DKC1 knockdown A549 and PC-9 cells. The mean telomere length was expressed as a ratio (T/S) of telomere repeat length (T) to the copy number of a single copy gene (S, β-Globin). The followings were the sequence of the PCR primers.

- T1: 5′-ACACTAAGGTTTGGGTTTGGGTTTG GGTTTGGGTTAGTGT-3'
- T2: 5′-TGTTAGGTATCC CTATCCCTATCCCTATCCCTATCCCTAACA-3'
- S1: 5′-CGGCGGCGGGCGGCGCGGGCTGGGCGGcttcatccacgttcaccttg-3'.
- S2: 5′-GCCCGGCCCGCCGCGCCCGTCCCGCCGgaggagaagtctgccgtt-3'.

### Cytochemical detection of senescence-associated-β-galactosidase activity

DKC1 stable knockdown A549 and PC-9 cells or control cells (0.5 million) were plated in 6-well plate. Chromogenic β-gal substrate X-gal (C0602, Beyotime biotechnology, shanghai, China) were used to stain the fixed cells for 8 h. The cells were washed with phosphate-buffered saline (PBS) and imaged by bright field microscopy.

### Subcutaneous xenograft

BABL/c female nude mice were obtained from Beijing Vital River Laboratory Animal Technology Company (Beijing) and randomly assigned into three groups (shCTL, shDKC1-1 and shDKC1-2). Each group contains 6 mice. 1.5 million DKC1 knockdown PC-9 cells or control cells were injected to 5–6 weeks-old mice subcutaneously. When the tumors were detectable, tumor length and width were measured every 3 days. Tumor volume was calculated by using the following formula: tumor volume = (length × width^2^)/2. After 30 days of injection, the tumor was removed and sent for immunohistochemistry analysis.

### Immunohistochemistry (IHC) and survival analysis

The paraffin-embedded tissues were sectioned and after rehydration, antigen retrieval and blocking, the slides were incubated with the following antibodies: anti-DKC1 (sc-373956, Santa Cruz Biotechnology, CA, USA), anti-Ki67 (550,609, BD Biosciences, Franklin, USA) and anti-γH2A.X (2595, Cell Signaling Technology, Boston, USA), at 4 °C, overnight. Next day, after the incubation of HRP (horseradish peroxidase)-conjugated secondary antibody for 1 h at room temperature, the DAB (3, 3′-diaminobenzidine) staining was used for detecting HRP signaling (KIHC-5, Proteintech Group, Chicago, USA). Then counterstain slides with hematoxylin. The integral optical density (IOD) of each target was measured by Image-Pro Plus. The DKC1 expression in human lung adenocarcinoma tissues was interpreted independently by two pathologists. Two characteristics were used for scoring the expression of DKC1 in slices: overall stain intensity (with possible values ranging from 0 to 3) and a score representing the percentage of tumor cells that were stained (1, 0–25%; 2, 25–50%; 3, 50–75% and 4, > 75%). An IHC score was then calculated by multiplying the values of the two characteristics. Based on IHC score, patients were divided into two groups: high DKC1 expression (IHC score $\geq$ 4) and low DKC1 expression (IHC score < 4) (Additional file 1: Table S1). Overall survival (OS) was estimated using the Kaplan–Meier method, and the log-rank test was used for statistical analysis.

### Quantitative RT-PCR (qRT-PCR)

Total RNA was isolated using TRIzol reagent (15,596,026, Thermo Fisher Scientific, Waltham, USA) according to the introductions. The cDNA is synthesized using One-step gDNA Removal and cDNA Synthesis Kit (AE311, TransGen Biotech, Beijing, China). The qRT-PCR were performed using the SYBR Green SuperMix (1708884AP, Bio-Rad, Hercules, USA) and normalized by the expression level of GAPDH. The sequences of the primers for qRT-PCR were listed as follows.

- DKC1 F: 5′-ATGGCGGATGCGGAAGTAAT-3'
- DKC1 R: 5′-CCACTGAGACGTGTCCAACT-3'
- TERC F: 5′-ACCCTAACTGAGAAGGGCGTA-3'
- TERC R: 5′-AATGAACGGTGGAAGGCGG-3'
- GAPDH F: 5′-GAAGGTGAAGGTCGGAGTC-3'
- GAPDH R: 5′-GAAGATGGTGATGGGATTTC-3'

### Statistics

GraphPad Prism was used to conduct the statistical analysis for most of the data unless indicated otherwise. Statistical significance was assessed by Student’s t-test or one-way ANOVA except when stated otherwise.

## Results

### High expression of DKC1 in LUAD predicts poor prognosis

**Fig. 1:**
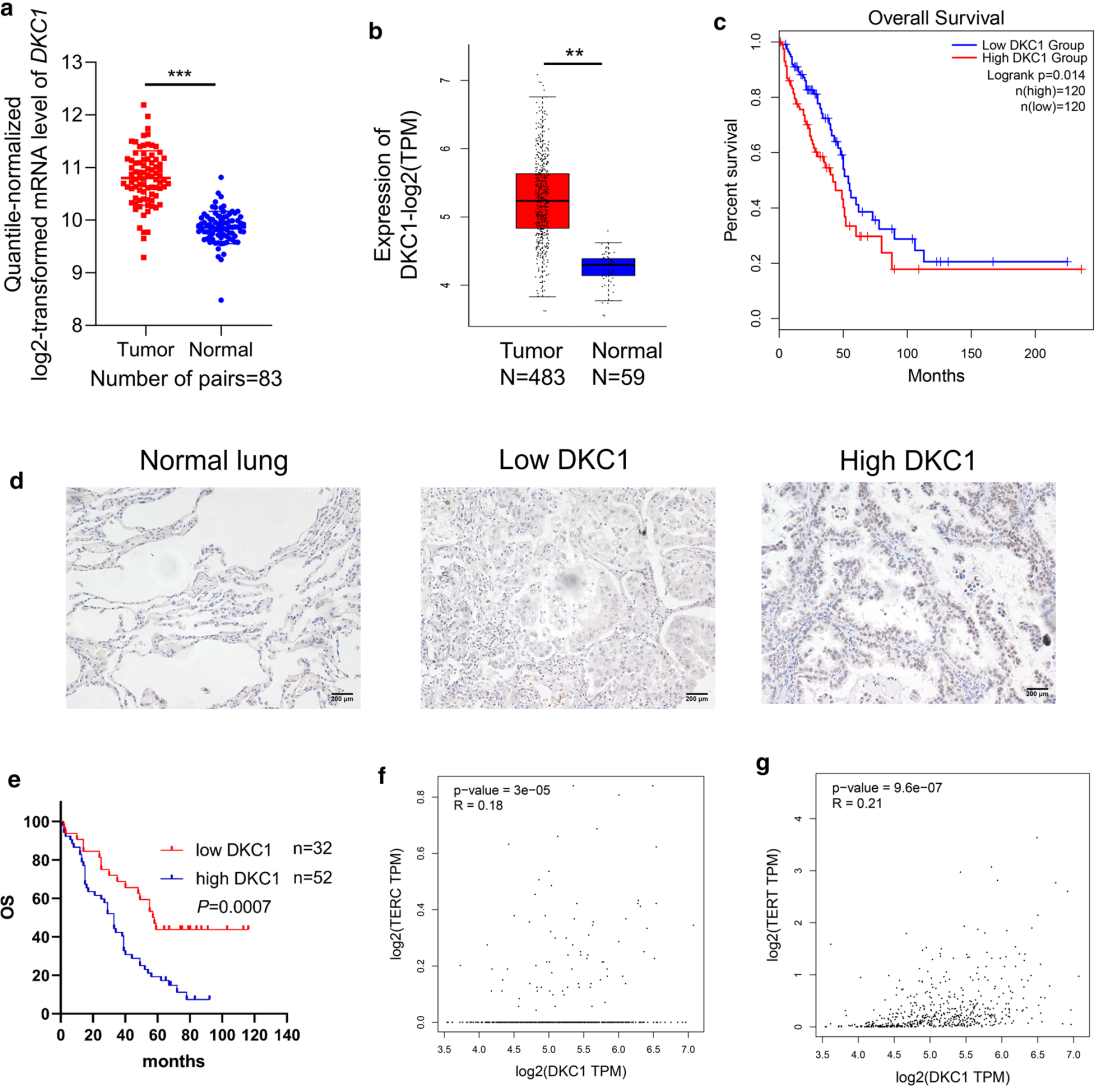
High expression of DKC1 in LUAD predicts poor prognosis. **a** The mRNA levels of *DKC1* in 83 LUAD tissues and paired adjacent normal tissues from the GEO database (GSE75037). **b** The mRNA levels of *DKC1* in LUAD and adjacent normal tissues from the TCGA database. **c** Kaplan–Meier analysis of overall survival (OS) of LUAD patients according to *DKC1* level from the TCGA LUAD database. **d, e** Immunohistochemistry (IHC) analysis of DKC1 expression in LUAD tissue microarray. **d** Representative IHC staining of DKC1 in the adjacent normal lung tissues, LUAD tissues with low or high DKC1 expression. Scale bar: 200 µm. **e** Kaplan–Meier analysis of OS in LUAD patients according to the DKC1 expression. **f, g** Correlation analysis between the *DKC1* level and the *TERC* abundance or the *TERT* abundance in the TCGA LUAD database. **P < 0.01, ***P < 0.001

We firstly examined DKC1 expression in LUAD tissues. The Gene Expression Omnibus (GEO) dataset (GSE75037) showed that compared with adjacent normal tissues, the 83 paired LUAD tissues show higher mRNA level of *DKC1* (Fig. 1a). In addition, The Cancer Genome Atlas (TCGA) database also revealed higher mRNA level of *DKC1* in LUAD tissues (N = 483) compared with the adjacent normal tissues (Fig. 1b). Patients with higher expression of *DKC1* from TCGA database had worse overall survival (OS) (Fig. 1c). To confirm the prognostic significance of high expression of DKC1 in LUAD patients, immunohistochemical (IHC) staining of DKC1 was performed on 84 LUAD tissues and 70 adjacent normal tissues (Additional file 1: Table S1). There is almost no DKC1 expression in adjacent normal tissues, and higher DKC1 expression in LUAD predicted worse OS (Fig. 1d, e). Besides, both *telomerase RNA component* (*TERC*) and *telomerase reverse transcriptase (TERT)* expression were positively correlated with *DKC1* expression in TCGA datasets (Fig. 1f, g).

### DKC1 accelerates LUAD cell proliferation

**Fig. 2:**
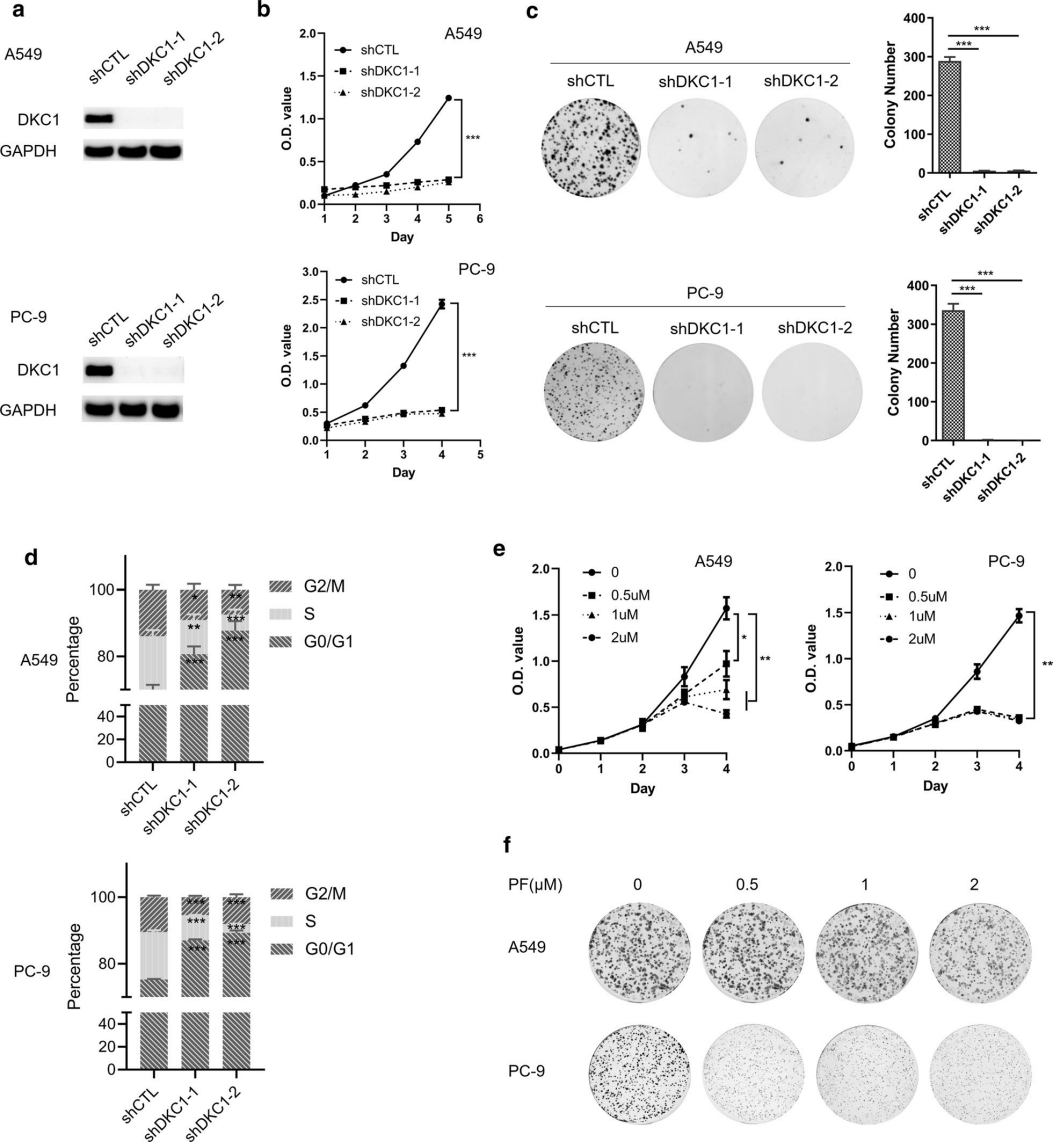
DKC1 knockdown inhibits LUAD cell proliferation. **a** Expression of DKC1 in LUAD cell lines (A549 and PC-9) after lentiviral transduction of shRNA against DKC1 or nonspecific control. **b** Cell growth curve of LUAD cell lines (A549 and PC-9) after DKC1 knockdown. **c** Colony formation assays of DKC1 knockdown and control cells. The left panel shows representative colony formation images; the right panel shows a bar graph of the number of colonies per well (mean ± SD). **d** Cell cycle assays of DKC1 knockdown A549 and PC-9 cells and control cells. **e, f** The effect of DKC1 inhibitor pyrazofurin on the growth (**e**) and colony formation (**f**) of A549 and PC-9 cells. *P < 0.05, **P < 0.01, ***P < 0.001

To investigate the function of DKC1 in LUAD, lentiviral transduction of DKC1 shRNAs were used to knockdown the DKC1 expression in two LUAD cell lines (A549 and PC-9). Both shRNAs against DKC1 could efficiently downregulate DKC1 protein levels in A549 and PC-9 (Fig. 2a). A549 and PC-9 cells with DKC1 silencing show decreased cell growth (Fig. 2b) and reduced colony number (Fig. 2c). In addition, A549 and PC-9 cells with DKC1 silencing showed cell cycle arrest at G1 phase and significantly decreased S phase and G2/M phase proportion (Fig. 2d). DKC1 inhibitor pyrazofurin (PF) also deceases cell growth and colony number of A549 and PC-9 cell lines (Fig. 2e, f). At the mean time, PF treatment (24 h) caused significant decrease of G2/M phase proportion (Additional file 1: Figure S1).

**Fig. 3:**
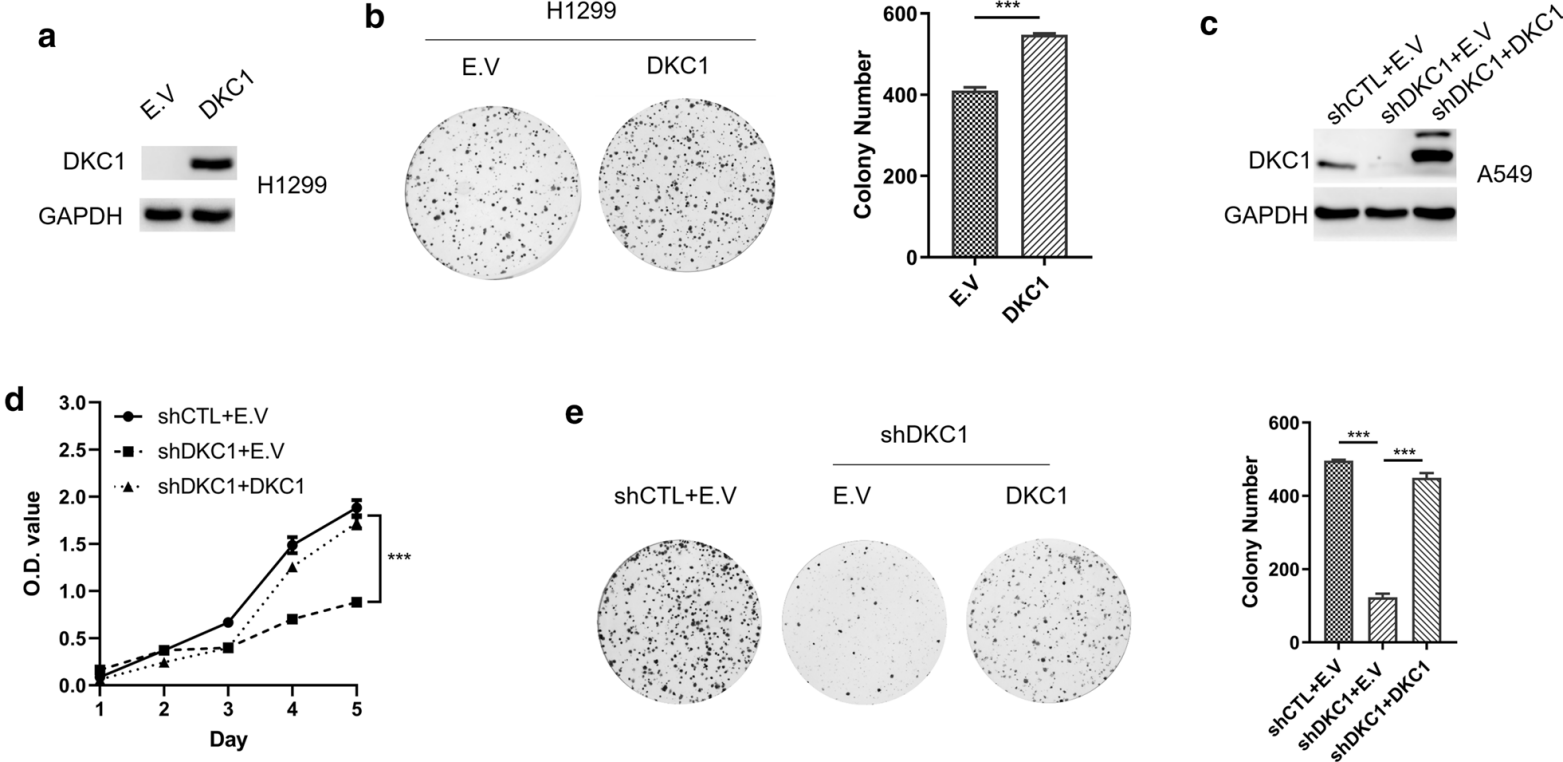
Ectopic expression of DKC1 accelerates LUAD cell proliferation. **a** Expression of DKC1 in NCI-H1299 cells after enforced expression of DKC1. **b** Colony formation assays of DKC1 overexpressed NCI-H1299 cells and control cells with a bar graph of the number of colonies per well (mean ± SD). **c** Immunoblot detecting DKC1 expression in control and DKC1 knockdown A549 cells with ectopic expression of DKC1. **d** Cell growth curve of DKC1 knockdown A549 cells with ectopic expression of DKC1. **e** Colony formation assays of DKC1 knockdown A549 cells with ectopic expression of DKC1 with a bar graph of the number of colonies per well (mean ± SD) on the right panel. ***P < 0.001

Next, lentiviral transduction of DKC1 in NCI-H1299 cells without endogenous DKC1 expression (Fig. 3a) significantly increased colony number (Fig. 3b). Furthermore, the successfully re-expressed DKC1 in DKC1 silenced A549 cells (Fig. 3c) could restore the cell growth and colony formation (Fig. 3d, e). Taken together, DKC1 accelerates cell cycle progression and promotes cell proliferation in LUAD cell lines.

### DKC1 downregulation induces telomere-related senescence and apoptosis

**Fig. 4:**
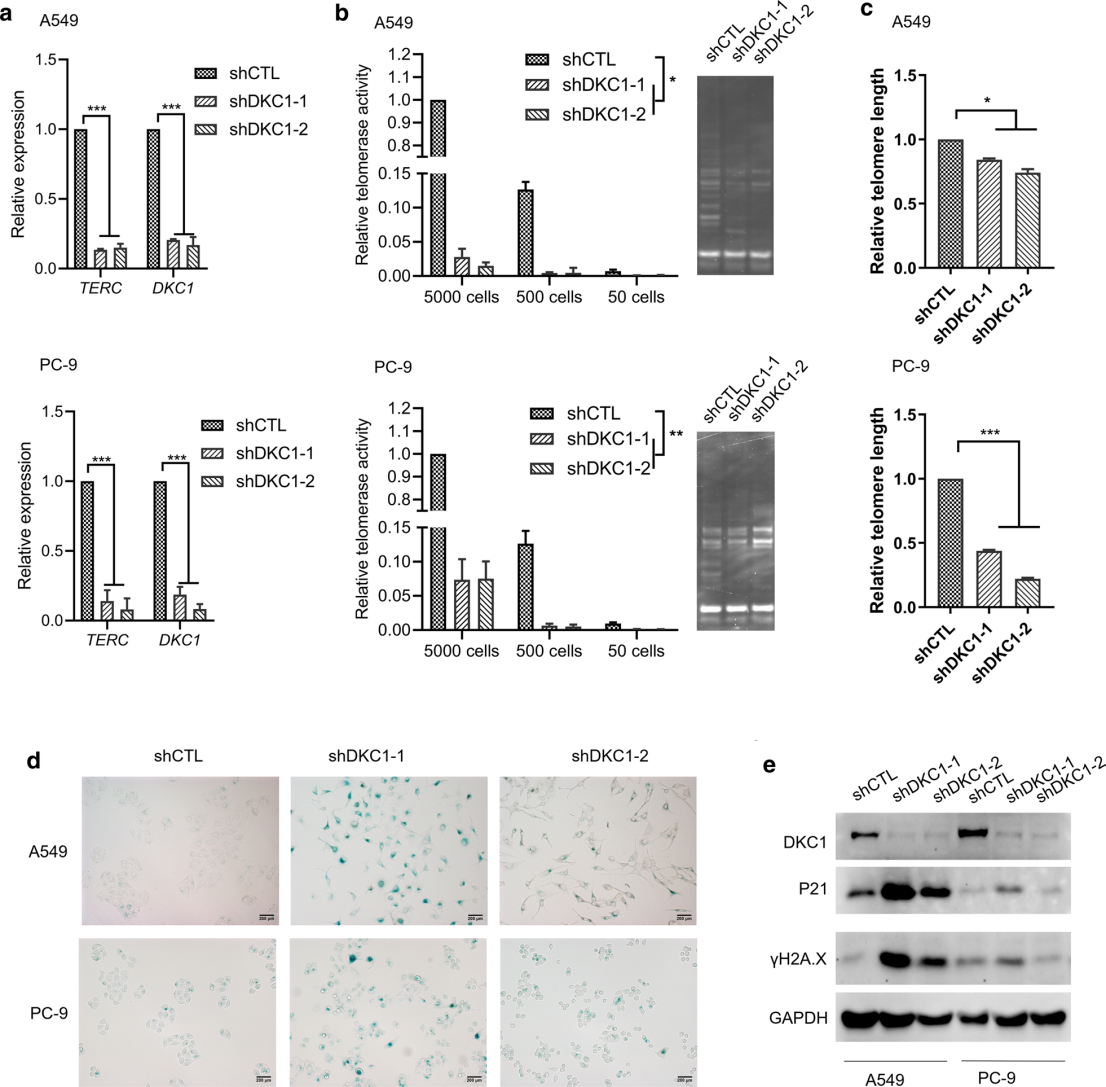
DKC1 downregulation induced telomere-related cell senescence and apoptosis. **a** qPCR detecting the abundance of *TERC* and *DKC1* in DKC1 knockdown A549 and PC-9 cells. **b** qPCR-based Telomerase Repeated Amplification Protocol (qTRAP) analyses for telomerase activity. The left panel shows the relative telomerase activity of DKC1 knockdown A549 and PC-9 cells (5000 cells, 500 cells and 50 cells). The right panel shows the gel image of TRAP assay on 5000 cells. **c** qPCR-based telomere length assessment of control and DKC1 silenced A549 and PC-9 cells. **d** Senescence-associated-β-galactosidase staining of control and DKC1 silenced A549 and PC-9 cells. Scale bar: 200 µm. **e** Immunoblot detecting DKC1, P21 and γH2A.X expression in control and DKC1 silenced A549 and PC-9 cells. *P < 0.05, **P < 0.01, ***P < 0.001

We further investigated the mechanism of DKC1 to promote cell proliferation. As DKC1 is one component of telomerase ribonucleoprotein, we firstly examined the effects of DKC1 downregulation on telomere in LUAD cell lines. Both the level of *TERC* and telomerase activity were significantly decreased in DKC1 silenced A549 and PC-9 cells (Fig. 4a, b). In accordance with this phenomenon, DKC1 downregulated A549 and PC-9 cells showed reduced length of telomere (Fig. 4c). In addition, we evaluated the effects of DKC1 knockdown on cell senescence and apoptosis. There was more senescence associated β-galactosidase positive cells in DKC1 silenced A549 and PC-9 cells (Fig. 4d), and the expression of cell senescence and apoptosis marker P21 and DNA damage marker γH2A.X are higher in A549 and PC-9 cells with DKC1 downregulation compared to control cells (Fig. 4e). These data suggest that DKC1 downregulation inhibits LUAD cell proliferation by inducing telomere-related cell senescence and apoptosis.

### DKC1 promotes LUAD cell proliferation in vivo

**Fig. 5:**
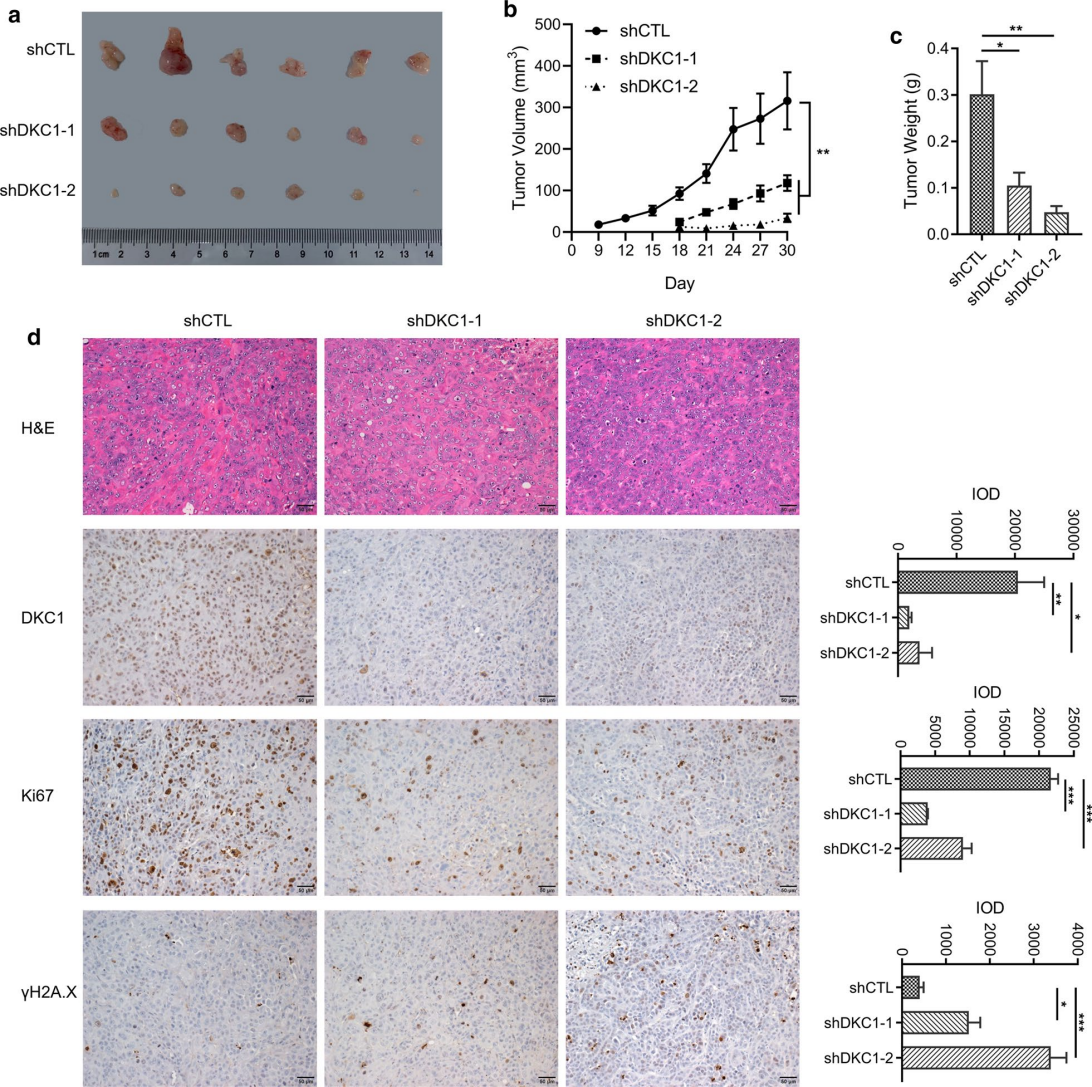
DKC1 knockdown inhibits LUAD proliferation in vivo*.* **a** Images of control and DKC1 knockdown PC-9 xenograft tumors obtained 30 days after subcutaneous inoculation. **b** Volume of control and DKC1 knockdown PC-9 xenograft tumors recorded at the indicated times. **c** Weight of control and DKC1 knockdown PC-9 xenograft tumors 30 days after inoculation. **d** H&E and IHC staining of DKC1, Ki67 and γH2A.X in control and DKC1 knockdown PC-9 xenograft tumor. The left panel shows the representative images and the right panel shows the quantitative analysis of DKC1, Ki67 or γH2A.X-positive cells according to integral optical density (IOD). Scale bar: 50 µm. *P < 0.05, **P < 0.01, ***P < 0.001

Finally, we validated the function of DKC1 in xenograft mice model. DKC1 silenced PC-9 cells and control cells were subcutaneously inoculated into the BALB/c nude mice. The tumor volume and weight were monitored and measured (Fig. 5a, c). The tumor growth is much slower in DKC1 silenced PC-9 cells compared to control cells (Fig. 5b). The detectable tumor for DKC1 silenced PC-9 cells emerged at day 18, which is significantly later than control cells (day 9) (Fig. 5b). At the end of the experiment, the tumor weight of DKC1 knockdown PC-9 cell group is lighter than the control group (Fig. 5c). Pathological analyses showed that the DKC1 knockdown tumor cells express less proliferation marker Ki67 compared to control cells (Fig. 5d), and there are more DNA damage marker γH2A.X positive cells in DKC1 knockdown tumors (Fig. 5d). Taken together, DKC1 accelerates LUAD cell proliferation in vitro and in vivo. Targeted inhibition of DKC1 in LUAD cells resulted in telomere shortening, cell senescence and apoptosis.

## Discussion

Cancer is the leading cause of death in the world and there is significantly increased survival rate in the early detected patients. Thus, it is extremely critical to explore new markers for precision diagnosis and prognosis as well as new targets for effective cancer treatment. And now, there are increasing number of molecular markers discovered for cancer diagnosis, prognosis and predicting therapy efficacy [17–21].

Telomeres play a critical role in preventing loss of essential genetic information during replication and distinguishing chromosome ends from DNA double-strand breaks, and ensure genome stability and integrity. Telomerase is a reverse transcriptase which adds telomeric repeats onto chromosome ends to maintain telomere length, and this process is carefully controlled in cells. More than 90% of human tumors including lung cancer exhibited reactivation of telomerase and short telomeres [22–24]. As there is almost no detectable telomerase activity in most normal tissues, inhibition of telomerase is an attractive strategy for cancer therapy. Several approaches have been developed to inhibit telomerase, including antisense oligodeoxynucleotides, hammerhead ribozymes, catalytic (hTERT) component and small molecule inhibitors [25, 26]. Some of them have entered the phase I and II clinical trials [25, 26]. For example, Imetelstat (GRN163L) acting as a direct telomerase RNA template antagonist has entered phase II clinical trials in advanced and metastatic non-small cell lung cancer (NSCLC) [27–29]. The efficacy of anti-telomerase therapy depends on the initial shortest telomere length which dictates the onset of telomere dysfunction [30]. As tumor cells exhibit short telomere and very little telomerase could maintain the shortest telomeres [31], highly potent inhibitors are needed. In this study, we found that high DKC1 expression is associated with LUAD progression and indicates poor prognosis. There is no significant difference in *Dkc1* expression between inflammatory non-transformed lung and normal lung of mice (Additional file 1: Figure S2). DKC1 downregulation inhibits telomerase activity, induces telomere length shorten in lung cancer cells. Targeted inhibition of DKC1 increased telomere-related senescence and apoptosis both in vitro and in vivo. Our research indicated that DKC1 is a promising target for telomerase-based therapies in lung cancer.

## Conclusions

In conclusion, our data suggest that high expression of DKC1 could act as a candidate marker for diagnosis and therapy target in lung adenocarcinoma by inducing telomere-related cell senescence and apoptosis.

## Supplementary Information

**Additional file 1: Figure S1.** The effect of PF on cell cycle in A549 and PC-9 cells. A549 cells (**a**) or PC-9 cells (**b**) were treated with the indicated concentration of PF for 24 h. The left panel shows the flow cytometric analysis of the indicated cells and the right panel shows the statistical analysis of the percentage of cells in G2/M phase of cell cycle. ***P < 0.001. **Figure S2.** *Dkc1* expression in inflammatory non-transformed lung. **a** The mRNA levels of *Dkc1* in lung tissues from smoking mice or sham mice for 6 weeks or 12 weeks (GSE12930). **b** The mRNA levels of *Dkc1* in lung tissues from mice with the indicated treatment (GSE102016). B[a]P: benzo(a)pyrene, LPS: lipopolysaccharides. ns: no significance. **Table S1.** The Patients’ clinical data and IHC score from LUAD tissue microarray

## Data Availability

Raw data of this study have been deposited in Research Data Deposit database (http://www.researchdata.org.cn), with the Approval Number as RDDB2021001094.

## Abbreviations

DKC1: Dyskerin pseudouridine synthase 1
LUAD: Lung adenocarcinoma
TERC: Telomerase RNA component
TERT: Telomerase reverse transcriptase
PF: Pyrazofurin
TRAP: Telomerase repeated amplification protocol
MET: Mesenchymal-to-epithelial transition

## References

[1] Bray F, Ferlay J, Soerjomataram I, Siegel RL, Torre LA, Jemal A (2018). Global cancer statistics 2018: GLOBOCAN estimates of incidence and mortality worldwide for 36 cancers in 185 countries. CA Cancer J Clin.

[2] Herbst RS, Morgensztern D, Boshoff C (2018). The biology and management of non-small cell lung cancer. Nature.

[3] Tulpule A, Bivona TG (2020). Acquired resistance in lung cancer. Annual Rev Cancer Biol.

[4] Coudray N, Ocampo PS, Sakellaropoulos T, Narula N, Snuderl M, Fenyö D (2018). Classification and mutation prediction from non-small cell lung cancer histopathology images using deep learning. Nat Med.

[5] Nelson ND, Bertuch AA (2012). Dyskeratosis congenita as a disorder of telomere maintenance. Mutat Res.

[6] Mason PJ, Bessler M (2011). The genetics of dyskeratosis congenita. Cancer Genet.

[7] Wong JMY, Collins K (2006). Telomerase RNA level limits telomere maintenance in X-linked dyskeratosis congenita. Genes Dev.

[8] Caton EA, Kelly EK, Kamalampeta R, Kothe U (2018). Efficient RNA pseudouridylation by eukaryotic H/ACA ribonucleoproteins requires high affinity binding and correct positioning of guide RNA. Nucleic Acids Res.

[9] Hou P, Shi P, Jiang T, Yin H, Chu S, Shi M (2019). DKC1 enhances angiogenesis by promoting HIF-1alpha transcription and facilitates metastasis in colorectal cancer. Br J Cancer..

[10] Miao FA, Chu K, Chen HR, Zhang M, Shi PC, Bai J (2019). Increased DKC1 expression in glioma and its significance in tumor cell proliferation, migration and invasion. Invest New Drugs..

[11] Ko E, Kim JS, Ju S, Seo HW, Chang Y, Kang JA (2018). Oxidatively modified protein-disulfide isomerase-associated 3 promotes dyskerin pseudouridine synthase 1-mediated malignancy and survival of hepatocellular carcinoma cells. Hepatology.

[12] Rocchi L, Barbosa AJ, Onofrillo C, Del Rio A, Montanaro L (2014). Inhibition of human dyskerin as a new approach to target ribosome biogenesis. PLoS ONE.

[13] Halappanavar S, Russell M, Stampfli MR, Williams A, Yauk CL (2009). Induction of the interleukin 6/ signal transducer and activator of transcription pathway in the lungs of mice sub-chronically exposed to mainstream tobacco smoke. BMC Med Genomics.

[14] Shi Q, Fijten RR, Spina D, Riffo Vasquez Y, Arlt VM, Godschalk RW (2017). Altered gene expression profiles in the lungs of benzo[a]pyrene-exposed mice in the presence of lipopolysaccharide-induced pulmonary inflammation. Toxicol Appl Pharmacol.

[15] Jiang S, Tang M, Xin H, Huang J (2017). Assessing telomerase activities in mammalian cells using the quantitative PCR-based telomeric repeat amplification protocol (qTRAP). Methods Mol Biol.

[16] Cawthon RM (2009). Telomere length measurement by a novel monochrome multiplex quantitative PCR method. Nucleic Acids Res.

[17] AbdelGhafar MT, Allam AA, Darwish S (2019). Serum HOX transcript antisense RNA expression as a diagnostic marker for chronic myeloid leukemia. Egypt J Haematol.

[18] El-Guindy DM, Wasfy RE, Abdel Ghafar MT, Ali DA, Elkady AM (2019). Oct4 expression in gastric carcinoma: association with tumor proliferation, angiogenesis and survival. J Egypt Natl Canc Inst.

[19] Abdel Ghafar MT, Gharib F, Abdel-Salam S, Elkhouly RA, Elshora A, Shalaby KH (2020). Role of serum Metadherin mRNA expression in the diagnosis and prediction of survival in patients with colorectal cancer. Mol Biol Rep.

[20] Abdel Ghafar MT, Gharib F, Al-Ashmawy GM, Mariah RA (2020). Serum high-temperature-required protein A2: A potential biomarker for the diagnosis of breast cancer. Gene Reports.

[21] Habib EM, Nosiar NA, Eid MA, Taha AM, Sherief DE, Hassan AE (2021). Circulating miR-146a expression predicts early treatment response to imatinib in adult chronic myeloid leukemia. J Investig Med.

[22] Fernandez-Garcia I, Ortiz-de-Solorzano C, Montuenga LM (2008). Telomeres and telomerase in lung cancer. J Thorac Oncol.

[23] Xu L, Li S, Stohr BA (2013). The role of telomere biology in cancer. Annu Rev Pathol.

[24] Günes C, Rudolph KL (2013). The role of telomeres in stem cells and cancer. Cell.

[25] White LK, Wright WE, Shay JW (2001). Telomerase inhibitors. Trends Biotechnol.

[26] Ruden M, Puri N (2013). Novel anticancer therapeutics targeting telomerase. Cancer Treat Rev.

[27] Mavroudis D, Bolonakis I, Cornet S, Myllaki G, Kanellou P, Kotsakis A (2006). A phase I study of the optimized cryptic peptide TERT(572y) in patients with advanced malignancies. Oncology.

[28] Bolonaki I, Kotsakis A, Papadimitraki E, Aggouraki D, Konsolakis G, Vagia A (2007). Vaccination of patients with advanced non–small-cell lung cancer with an optimized cryptic human telomerase reverse transcriptase peptide. J Clin Oncol.

[29] Chiappori AA, Kolevska T, Spigel DR, Hager S, Rarick M, Gadgeel S (2015). A randomized phase II study of the telomerase inhibitor imetelstat as maintenance therapy for advanced non-small-cell lung cancer. Ann Oncol.

[30] Hemann MT, Strong MA, Hao L-Y, Greider CW (2001). The shortest telomere, not average telomere length, is critical for cell viability and chromosome stability. Cell.

[31] Ouellette MM, Liao M, Herbert BS, Johnson M, Holt SE, Liss HS (2000). Subsenescent telomere lengths in fibroblasts immortalized by limiting amounts of telomerase. J Biol Chem.

