# Supplementary material for: Inhibition of DKC1 induces telomere-related senescence and apoptosis in lung adenocarcinoma
Source: J Transl Med. 2021 Apr 20;19:161. doi: 10.1186/s12967-021-02827-0 (PMC8056518; doi:10.1186/s12967-021-02827-0)
Supplement: Supplementary file 1 — Additional file 1: Figure S1. The effect of PF on cell cycle in A549 and PC-9 cells. A549 cells (a) or PC-9 cells (b) were treated with the indicated concentration of PF for 24 h. The left panel shows the flow cytometric analysis of the indicated cells and the right panel shows the statistical analysis of the percentage of cells in G2/M phase of cell cycle. ***P < 0.001. Figure S2. Dkc1 expression in inflammatory non-transformed lung. a The mRNA levels of Dkc1 in lung tissues from smoking mice or sham mice for 6 weeks or 12 weeks (GSE12930). b The mRNA levels of Dkc1 in lung tissues from mice with the indicated treatment (GSE102016). B[a]P: benzo(a)pyrene, LPS: lipopolysaccharides. ns: no significance. Table S1. The Patients’ clinical data and IHC score from LUAD tissue microarray [file 12967_2021_2827_MOESM1_ESM.docx]

**Additional file 1**

**Inhibition of DKC1 induces telomere-related senescence and apoptosis in lung adenocarcinoma**

Guangyan Kan^1^, Ziyang Wang^1^, Chunjie Sheng^1^, Chen Yao^1^, Yizhi Mao^1^, Shuai Chen^1^*

This Additional file include:

1. Two Additional figures
2. One Additional table

**Additional file 1: Figures**

**
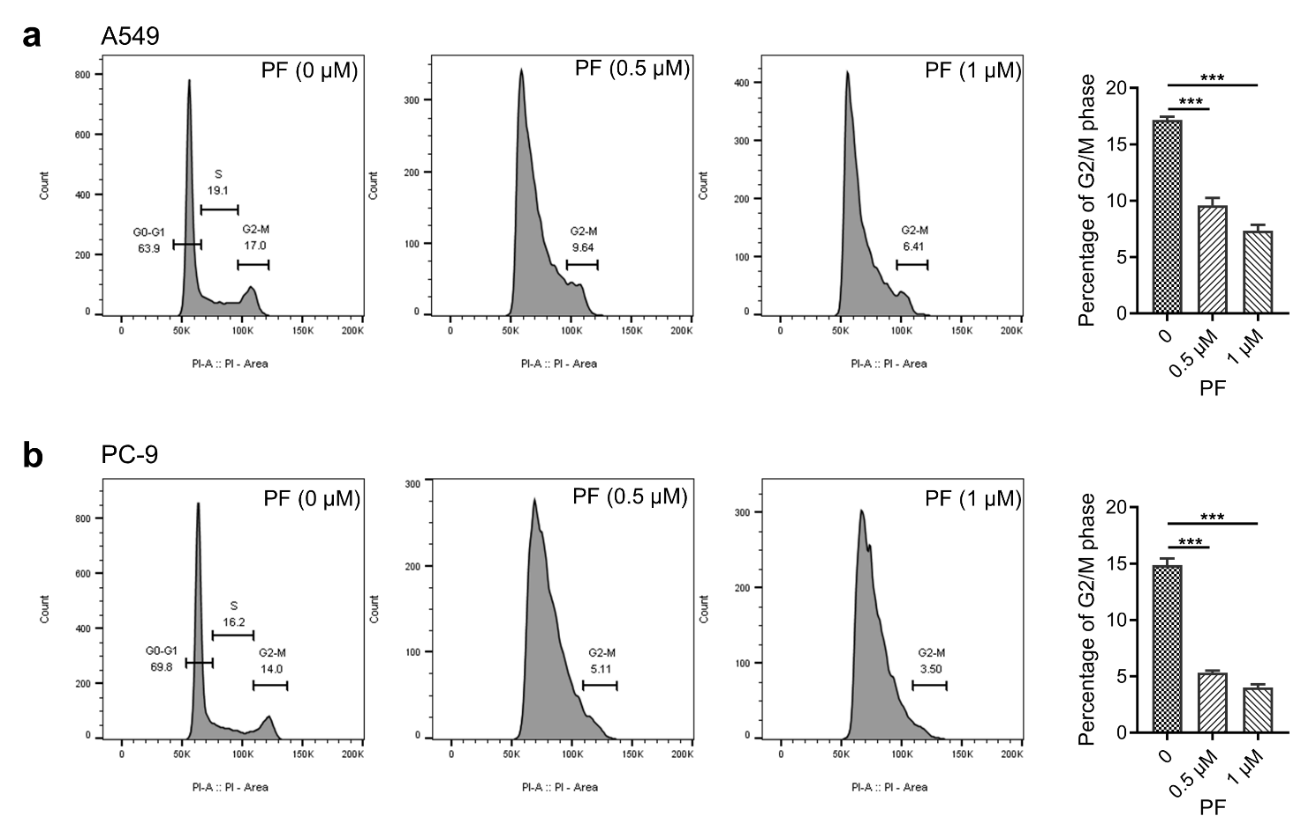
**

**Figure S1.** The effect of PF on cell cycle in A549 and PC-9 cells. A549 cells (**a**) or PC-9 cells (**b**) were treated with the indicated concentration of PF for 24 h. The left panel shows the flow cytometric analysis of the indicated cells and the right panel shows the statistical analysis of the percentage of cells in G2/M phase of cell cycle. ****P*<0.001.


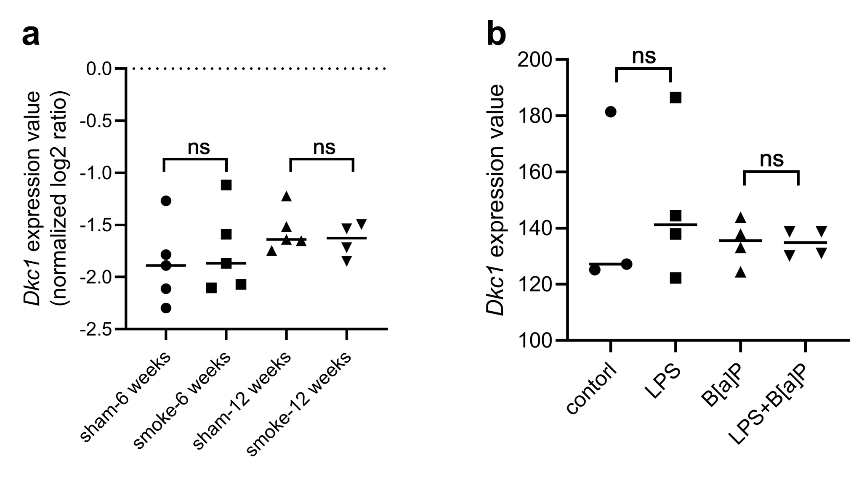


**Figure S2.** *Dkc1* expression in inflammatory non-transformed lung. **a** The mRNA levels of *Dkc1* in lung tissues from smoking mice or sham mice for 6 weeks or 12 weeks (GSE12930). **b** The mRNA levels of *Dkc1* in lung tissues from mice with the indicated treatment (GSE102016). B[a]P: benzo(a)pyrene, LPS: lipopolysaccharides. ns: no significance.

**Additional file 1: Table**

**Table S1.** The Patients’ clinical data and IHC score from LUAD tissue microarray

| **Tissue ID** | **Tissues Obtained** | **Surgery Time** | **Vital Status** | **Follow-up Time** | **Survival Time (month)** | **DKC1 IHC score** |
| --- | --- | --- | --- | --- | --- | --- |
| E05A0032 | Tumor and adjacent normal tissues | 2004/9/30 | dead | 2011/3/13 | 78 | 4.00 |
| E05A0036 | Tumor and adjacent normal tissues | 2004/10/11 | dead | 2007.12.08 | 38 | 12.00 |
| E05A0046 | Tumor and adjacent normal tissues | 2004/11/26 | dead | 2008/12/10 | 49 | 12.00 |
| E05A0060 | Tumor and adjacent normal tissues | 2004/12/30 | alive | 2014/8/20 | 116 | 3.00 |
| E05A0081 | Tumor and adjacent normal tissues | 2005/3/8 | alive | 2014/8/20 | 113 | 3.00 |
| E05A0104 | Tumor and adjacent normal tissues | 2005/5/17 | dead | 2008/2/6 | 33 | 4.00 |
| E05A0123 | Tumor | 2005/6/30 | dead | 2007/3/22 | 21 | 8.00 |
| E05A0142 | Tumor and adjacent normal tissues | 2005/11/7 | dead | 2009/2/23 | 39 | 4.00 |
| E05A0146 | Tumor and adjacent normal tissues | 2005/11/14 | dead | 2008/9/28 | 34 | 4.00 |
| E05A0149 | Tumor and adjacent normal tissues | 2005/11/23 | dead | 2009.02.13 | 39 | 4.00 |
| E05A0175 | Tumor and adjacent normal tissues | 2006/1/3 | alive | 2014/8/20 | 103 | 3.00 |
| E05A0182 | Tumor and adjacent normal tissues | 2006/2/27 | dead | 2007.05.12 | 15 | 4.00 |
| E05A0195 | Tumor and adjacent normal tissues | 2006/8/24 | dead | 2011/3/25 | 55 | 1.00 |
| E05A0201 | Tumor and adjacent normal tissues | 2006/8/31 | dead | 2011/10/3 | 62 | 4.00 |
| E05A0208 | Tumor and adjacent normal tissues | 2006.11 | dead | 2009.08.25 | 33 | 4.00 |
| E05A0209 | Tumor and adjacent normal tissues | 2006/11/13 | dead | 2008.01.04 | 14 | 8.00 |
| E05A0210 | Tumor and adjacent normal tissues | 2006/11/13 | dead | 2010.12.24 | 49 | 4.00 |
| E05A0211 | Tumor and adjacent normal tissues | 2006.11 | dead | 2007.12.18 | 13 | 8.00 |
| E05A0252 | Tumor and adjacent normal tissues | 2006.12 | alive | 2014/8/20 | 92 | 4.00 |
| E05A0255 | Tumor and adjacent normal tissues | 2007.1 | alive | 2014/8/20 | 91 | 3.00 |
| E05A0270 | Tumor and adjacent normal tissues | 2007/1/12 | alive | 2014/8/20 | 91 | 2.00 |
| E05A0272 | Tumor and adjacent normal tissues | 2007/1/22 | dead | 2008/4/22 | 15 | 12.00 |
| E05A0279 | Tumor and adjacent normal tissues | 2007.3 | dead | 2008/8/21 | 17 | 8.00 |
| E05A0280 | Tumor | 2007.3 | dead | 2009/12/22 | 33 | 8.00 |
| E05A0289 | Tumor and adjacent normal tissues | 2007.3 | dead | 2012/2/12 | 59 | 1.00 |
| E05A0310 | Tumor and adjacent normal tissues | 2007/4/4 | dead | 2011/4/25 | 48 | 3.00 |
| E05A0317 | Tumor | 2007/4/5 | dead | 2007/7/20 | 3 | 4.00 |
| E05A0318 | Tumor | 2007/4/6 | dead | 2009/7/31 | 27 | 4.00 |
| E05A0321 | Tumor and adjacent normal tissues | 2007.4 | dead | 2010.12.13 | 44 | 4.00 |
| E05A0332 | Tumor and adjacent normal tissues | 2007.5 | alive | 2014/8/20 | 87 | 1.00 |
| E05A0333 | Tumor and adjacent normal tissues | 2007.5 | dead | 2009/6/27 | 25 | 4.00 |
| E05A0347 | Tumor | 2007.6 | dead | 2012/2/4 | 56 | 8.00 |
| E05A0349 | Tumor | 2007.5 | dead | 2012/12/22 | 67 | 4.00 |
| E05A0353 | Tumor | 2007.7 | dead | 2009.12.09 | 29 | 4.00 |
| E05A0355 | Tumor and adjacent normal tissues | 2007.7 | dead | 2008/11/18 | 16 | 4.00 |
| E05A0359 | Tumor and adjacent normal tissues | 2007.8 | dead | 2008/2/24 | 6 | 4.00 |
| E05A0364 | Tumor and adjacent normal tissues | 2007.9 | dead | 2008/11/16 | 14 | 2.00 |
| E05A0369 | Tumor and adjacent normal tissues | 2007/5/25 | dead | 2008.06.17 | 13 | 8.00 |
| E05A0392 | Tumor and adjacent normal tissues | 2007/8/1 | dead | 2010/12/15 | 40 | 8.00 |
| E05A0396 | Tumor | 2007/8/6 | alive | 2014/8/20 | 84 | 3.00 |
| E05A0399 | Tumor and adjacent normal tissues | 2007/8/23 | dead | 2012/5/10 | 57 | 1.00 |
| E05A0403 | Tumor and adjacent normal tissues | 2007.9 | alive | 2014/8/20 | 83 | 4.00 |
| E05A0426 | Tumor and adjacent normal tissues | 2007.12 | dead | 2010.09.17 | 33 | 8.00 |
| E05A0448 | Tumor and adjacent normal tissues | 2007.12 | dead | 2011.04.11 | 40 | 1.00 |
| E05A0449 | Tumor and adjacent normal tissues | 2007.12 | dead | 2009.03.24 | 15 | 8.00 |
| E05A0450 | Tumor | 2007.12 | alive | 2014/8/20 | 80 | 1.00 |
| E05A0456 | Tumor | 2007/12/14 | dead | 2013/12/6 | 72 | 6.00 |
| E05A0463 | Tumor and adjacent normal tissues | 2008.1 | dead | 2010/12/11 | 35 | 1.00 |
| E05A0464 | Tumor and adjacent normal tissues | 2008.1 | alive | 2014/8/20 | 79 | 2.00 |
| E05A0471 | Tumor and adjacent normal tissues | 2008.2 | dead | 2010/3/22 | 25 | 1.00 |
| E05A0475 | Tumor and adjacent normal tissues | 2008.3 | dead | 2012/4/2 | 49 | 3.00 |
| E05A0482 | Tumor and adjacent normal tissues | 2008.3 | dead | 2011/6/3 | 39 | 12.00 |
| E05A0486 | Tumor and adjacent normal tissues | 2008.3 | dead | 2013/1/13 | 58 | 2.00 |
| E05A0502 | Tumor and adjacent normal tissues | 2008/3/3 | dead | 2008/10/18 | 7 | 4.00 |
| E05A0519 | Tumor and adjacent normal tissues | 2008.5 | alive | 2014/8/20 | 75 | 3.00 |
| E05A0520 | Tumor and adjacent normal tissues | 2008.6 | dead | 2008/7/14 | 1 | 4.00 |
| E05A0523 | Tumor and adjacent normal tissues | 2008.6 | alive | 2014/8/20 | 74 | 3.00 |
| E05A0537 | Tumor and adjacent normal tissues | 2008/5/12 | dead | 2012/9/13 | 52 | 4.00 |
| E05A0546 | Tumor and adjacent normal tissues | 2008/6/25 | dead | 2012/12/4 | 54 | 8.00 |
| E05A0550 | Tumor and adjacent normal tissues | 2008.7 | dead | 2009/9/14 | 14 | 1.00 |
| E05A0565 | Tumor and adjacent normal tissues | 2008.8 | dead | 2009/10/18 | 12 | 8.00 |
| E05A0575 | Tumor | 2008/7/8 | dead | 2009/7/4 | 12 | 4.00 |
| E05A0580 | Tumor and adjacent normal tissues | 2008/7/16 | dead | 2008/10/15 | 3 | 1.00 |
| E05A0584 | Tumor and adjacent normal tissues | 2008.10 | dead | 2010/3/5 | 15 | 8.00 |
| E05A0592 | Tumor and adjacent normal tissues | 2008.12 | dead | 2009/2/4 | 2 | 6.00 |
| E05A0594 | Tumor and adjacent normal tissues | 2008.12 | dead | 2009/2/4 | 2 | 0.00 |
| E05A0595 | Tumor and adjacent normal tissues | 2008.12 | dead | 2011/5/29 | 29 | 4.00 |
| E05A0596 | Tumor and adjacent normal tissues | 2008.12 | alive | 2014/8/20 | 68 | 8.00 |
| E05A0616 | Tumor | 2008.12 | dead | 2009/2/11 | 2 | 4.00 |
| E05A0622 | Tumor and adjacent normal tissues | 2008/11/27 | dead | 2012/2/2 | 39 | 6.00 |
| E05A0627 | Tumor and adjacent normal tissues | 2008/12/9 | dead | 2011/1/16 | 25 | 3.00 |
| E05A0633 | Tumor and adjacent normal tissues | 2008/12/25 | alive | 2014/8/20 | 68 | 4.00 |
| E05A0639 | Tumor and adjacent normal tissues | 2009.1 | dead | 2010/4/30 | 15 | 4.00 |
| E05A0640 | Tumor and adjacent normal tissues | 2009.1 | alive | 2014/8/20 | 67 | 1.00 |
| E05A0648 | Tumor and adjacent normal tissues | 2009/2/12 | dead | 2009.12.14 | 10 | 1.00 |
| E05A0659 | Tumor and adjacent normal tissues | 2009.3 | dead | 2011/9/17 | 30 | 0.00 |
| E05A0663 | Tumor and adjacent normal tissues | 2008.4 | dead | 2010/4/6 | 24 | 3.00 |
| E05A0671 | Tumor and adjacent normal tissues | 2009/3/13 | alive | 2014/8/20 | 65 | 8.00 |
| E05A0677 | Tumor and adjacent normal tissues | 2009/4/8 | alive | 2014/8/20 | 64 | 2.00 |
| E05A0682 | Tumor and adjacent normal tissues | 2009.3 | dead | 2013/10/16 | 55 | 3.00 |
| E05A0699 | Tumor | 2009.4 | dead | 2009.12.07 | 8 | 4.00 |
| E05A0702 | Tumor and adjacent normal tissues | 2009.4 | alive | 2014/8/20 | 64 | 2.00 |
| E05A0703 | Tumor | 2009.4 | dead | 2011/9/16 | 29 | 4.00 |
| E05A0726 | Tumor and adjacent normal tissues | 2009.6 | alive | 2014/8/20 | 62 | 4.00 |
